# Single-cell pH imaging and detection for pH profiling and label-free rapid identification of cancer-cells

**DOI:** 10.1038/s41598-017-01956-1

**Published:** 2017-05-11

**Authors:** Hui Hou, Yangyang Zhao, Chuanping Li, Minmin Wang, Xiaolong Xu, Yongdong Jin

**Affiliations:** 1 State Key Laboratory of Electroanalytical Chemistry, Changchun Institute of Applied Chemistry, Chinese Academy of Sciences, Changchun, 130022 Jilin China; 2 University of Chinese Academy of Sciences, Beijing, 100049 China

## Abstract

Single-cell pH-sensing and accurate detection and label-free fast identification of cancer-cells are two long-standing pursuits in cell and life science, as intracellular pH plays a crucial role in many cellular events and fates, while the latter is vital for early cancer theranostics. Numerous methods based on functionalized nanoparticles and fluorescence probes have been developed for cell pH-sensing, but are often hindered for single-cell studies by their main drawbacks of complicated probe preparation and labeling, low sensitivity and poor reproducibility. Here we report a simple and reliable method for single-cell pH imaging and sensing by innovative combined use of UV-Vis microspectroscopy and common pH indicators. Accurate and sensitive pH detection on single-cell or sub-cell level with good reproducibility is achieved by the method, which enables facile single-cell pH profiling and label-free rapid identification of cancer-cells (due to distinguishable intracellular pH levels) for early cancer diagnosis, and may open a new avenue for pH-related single-cell studies.

## Introduction

Intracellular pH value is an essential factor that regulates many cellular behaviors, such as many pathological and physiological processes, the function of many organelles as well as enzyme activity and protein degradation^1, 2^. For a typical mammalian cell, the intracellular pH value can vary from 4.7 in lysosome to 8.0 in mitochondria. Disruptive variation in the intracellular pH may lead to functional disorder of the organelles^3, 4^. Usually abnormal intracellular pH value is a characteristic of many common diseases such as Alzheimer’s disease, stroke, and cancer^4, 5^. As we know, cancer cells are typically characterized by uncontrolled cell growth and abnormal acidic intracellular pH values^6, 7^. Up to now, various functionalized nanoparticles^6, 8^ and fluorescence indicators^5, 9–12^ have been developed for measuring intracellular pH. However, the synthesis processes of such kind of functionalized nanoparticles are complicated and usually needs labeling, and the fluorescence imaging method used for living-cell imaging always suffers from non-ignorable background signal, photobleaching and instability. These disadvantages limit their use for accurate single-cell pH detection and studies. Therefore, there is still an urgent demand to develop a simple and reliable method that is effective for sensitive detection and monitoring of intracellular pH change on single-cell or sub-cell level, which is crucial for studying cellular metabolisms and further gaining insights into pH-dependent physiological and pathological processes^13–16^.

**Figure 1:**
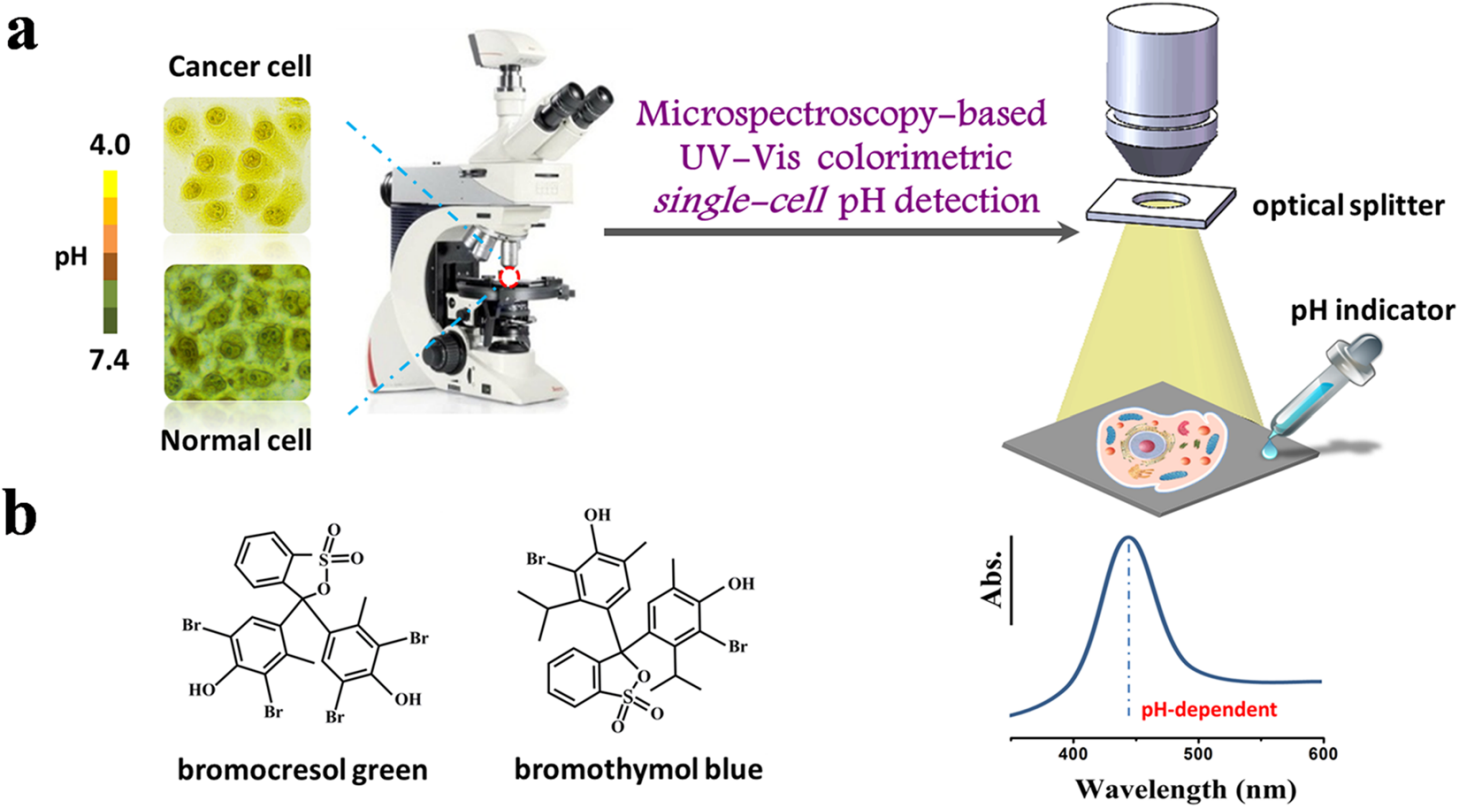
Schematic of colorimetric single-cell pH imaging and detection with two pH indicators. (**a**) Schematic of single-cell pH imaging and detection by combined use of bright-field microscope-based UV-Vis microspectroscopy and common pH indicators. A selected area single-cell absorption spectrum is collected by an optical microscope equipped with a portable spectrometer and an optical splitter with a small collecting area. (**b**) The structures of two pH indicators used in this study.

Here, we developed a simple and effective colorimetric imaging method for single-cell pH sensing and accurate detection by combining bright-field microscope-based UV-Vis microspectroscopy and common pH indicators, as schematically illustrated in Fig. 1a. Two commonly used pH indicators, bromothymol blue and bromocresol green (chemical structures shown in Fig. 1b) are selected for living-cell pH sensing in this study. They have partially different pH sensing ranges (Fig. S1) that basically cover intracellular pH ranges of normal or cancerous cells. Typically, bromocresol green is a pH indicator mostly used in applications that require measuring substances which have a relatively acidic pH (pH range: ~3.8–5.4). It will ionize to give the monoanionic form (yellow), and then further deprotonates at higher pH to give the dianionic form (blue). The color of bromocresol green/PBS solutions varied from bright yellow to deep blue as the pH increased from 3.0 to 7.5 (pH values of the PBS solution were adjusted by HCl) (Fig. S1A). Bromothymol blue (also known as bromothymolsulfonephthalein and BTB), which acts as a weak acid in solution, is a pH indicator mostly used for measuring substances which have a relatively neutral pH (near 7). It can thus be in protonated or deprotonated form, appearing yellow or blue, respectively. It is bluish green in neutral solution. The presence of one moderate electron withdrawing group (bromine atom) and two moderate donating groups (alkyl substituents) are responsible for bromothymol blue’s active indication range of pH between 6.0 and 7.6. The color of bromothymol blue/ PBS solutions varied from bright yellow to deep blue as the pH increased from 4.0 to 7.5 (Fig. S1B). When incubated and interacted with the pH indicators the cancer cells under bright-field microscopy exhibit yellow and bright color due to their acidic extra- and intra-cellular pH values^6, 7^; While the healthy mammalian cells present blue and dark color as their intracellular environment are close to neutral^13^. This forms the basis for easy visual identification of cancer cells from normal healthy cells by the cell pH imaging methodology.

## Results

### Living-cell bright-field pH imaging and colorimetric identification of cancer cells with pH indicators

**Figure 2:**
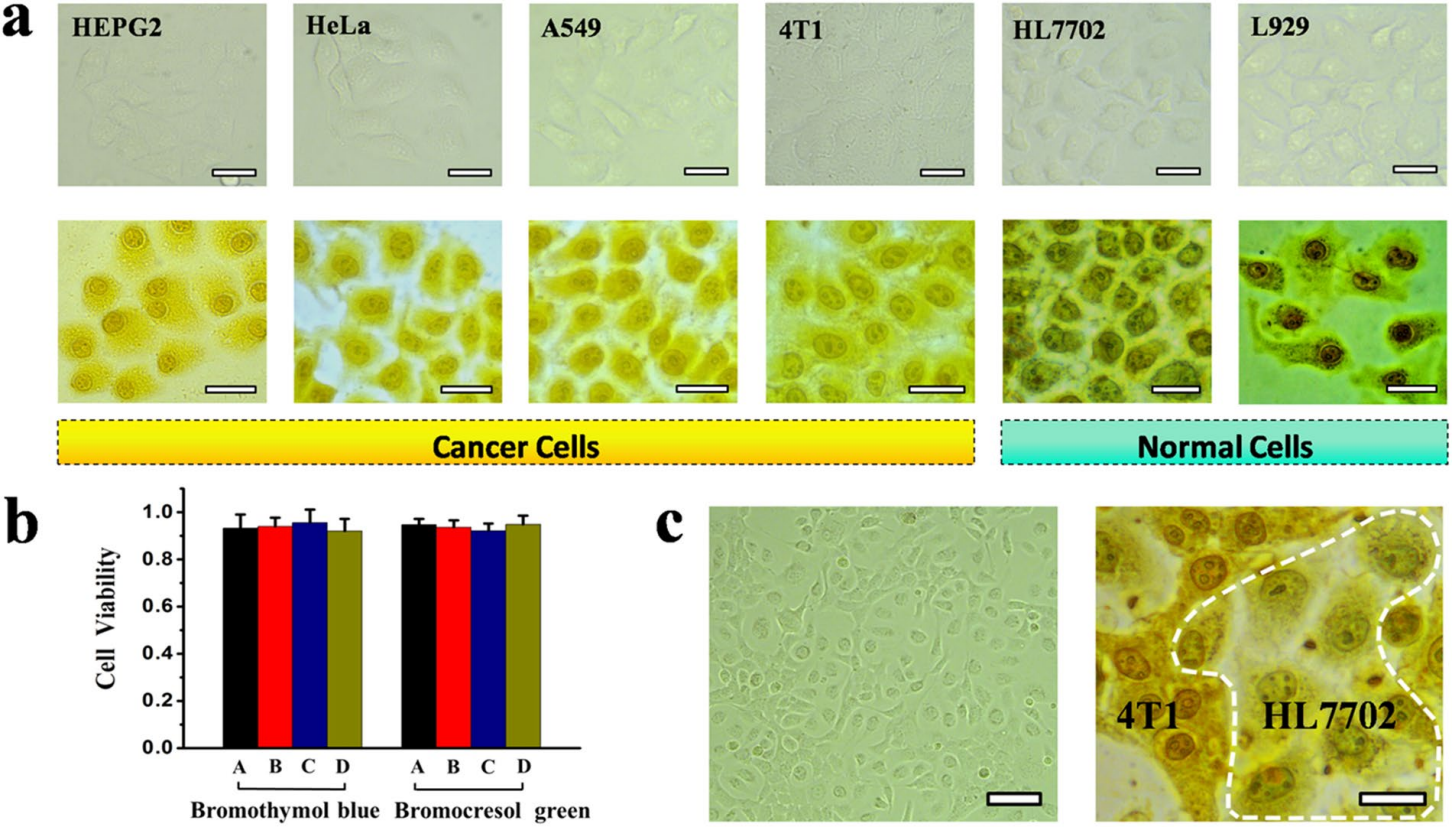
Visual identification and screening of cancer cells from normal cells and cytotoxicity assays. (**a**) Bright-field optical microscope images of cancerous HepG2, HeLa, A549, 4T1, normal HL7702 and L929 cells before and after 5 min incubation with 1 mg mL^−1^ of bromothymol blue, respectively; All scale bars: 20 μm. (**b**) Cytotoxicity assays for the A549, HeLa, HL7702, and L929 cells after the incubation with bromothymol blue or bromocresol green. A, B, C, D under the X-coordinate represent A549, HeLa, HL7702, and L929 cells, respectively. (**c**) Bright-field microscopy images of cocultured cancerous 4T1 and normal HL7702 cells before (left, scale bar: 50 μm) and after (right, scale bar: 15 μm) the treatment with bromocresol green. The cells within the white dotted line are identified as HL7702 cell.

We first closely inspected the feasibility of the two pH indicators for intracellular pH sensing and imaging. In a typical experiment, cells attached to the plate were simply treated with a 1 mg mL^−1^ of bromothymol blue or bromocresol green solution and incubated briefly for ~5 min. After washing with 20% ethanol aqueous solution for three times to remove excess extracellular pH indicators, the plate was then submitted for microscopy imaging. Typical four tumor cell lines (HepG2, HeLa, A549, and 4T1) and two normal cell lines (HL7702 and L929) were tested. Here, in order to check the potential damage of cells after the treatment, we randomly chose a tumor cell line (HeLa) and a normal cell line (L929), and treated them with 20% ethanol for 10 min (which is much longer than the time we used in the experiment), then the cells were stained with trypan blue dye. All of the HeLa (A) and L929 (B) cells were clean but not dyed blue, which means that almost all of the cells remain alive after the treatment (Fig. S2). Therefore, the 20% ethanol treatment did not have a detectable effect on the cell viability. Figure 2a shows representative bright-field microscopy images of the six kind of cells before (the first row) and after (the second row) the treatment with bromothymol blue. The outlines and subcellular structures of all tested six cell lines (without the treatment with bromothymol blue) are very obscure. After the treatment with bromothymol blue the cell outlines and some subcellular structures are seen clearly. It therefore reveals that the bromothymol blue molecules can enter easily into the cells and are uniformly distributed, rendering it promising as an effective.

Cell-staining agent for potential routine living-cell imaging applications. All the four kinds of cancer cells were dyed with bright yellow due to their acidic extra- and intra-cellular pH environment (Fig. 2a)^6, 7, 16–18^. But for different cancerous cell lines, there is an observable color difference in bright-field microscopy images, which is indicative of somewhat different acidic intracellular environments of the cells. For the healthy normal HL7702, L929 cells, the degree of coloration was significantly deeper than that of cancer cells. The nucleus and cytoplasm of the normal cells were stained with dark blue as their intracellular environments are close to neutral^13^. The normal cells and cancer cells therefore can be easily distinguished due to pronounced imaging difference, even if the (intracellular) blue color was partly overlapped by the (background) bright yellow arising outside the cells caused by cell surface-adsorpted or extracellular remaining a small amount of bromothymol blue molecules. We further tested the feasibility of bromocresol green for intracellular pH sensing and imaging. The bromocresol green showed similar results as that of bromothymol blue (Fig. S3). As compared with bromothymol blue the pH indication range of the bromocresol green is relatively narrow (pH range: ~3.8–5.4), we didn’t apply it for subsequent accurate pH detection of single cell. Incidentally, we also tried to use the pH indicators methyl red and thymol blue for the intracellular pH measurements, but all failed (Fig. S4). The results showed that the cells were still colorless after the treatment, which means that the two indicators (methyl red and thymol blue) are hard to enter the cells. Otherwise, the cells will represent a certain color (red, yellow or blue) due to their intracellular pH environment.

### Cell Viability Study

Next, the cytotoxicity of the bromothymol blue and bromocresol green was carefully checked by the standard 3-(4,5-dimethyl-2-thiazolyl)-2,5 diphenyltetrazolium bromide (MTT) assay^19^. As illustrated in Fig. 2b, the cell viability of all four cell lines tested (A549, HeLa, HL7702, and L929 cells) are not obviously affected after 24 h incubation with bromothymol blue or bromocresol green at concentration of 1 mg mL^−1^, indicating that the two pH indicators are safe for cells. Therefore, we applied the two pH indicators in this study for further visual discrimination of cancer-cells (from cocultured normal cells) and colorimetric accurate single-cell pH detection by combined use of UV-Vis microspectroscopy.

### Bright-field imaging identification of cocultured cancerous and normal cells

To further check the feasibility of the colorimetric imaging method for potential clinical application, a coculture of cancerous 4T1 and normal HL7702 cells (ratio of 1:1), acting as a simulated clinical cell sample in this study, was prepared and tested. Figure 2c shows the bright-field microscopy images of cocultured 4T1 and HL7702 cells before and after the treatment of bromocresol green. The HL7702 cells are dispersively distributed in the networked structure of 4T1 cells when they are cocultured. More importantly, the tumor cells can still be easily visually identified from cocultured normal healthy cells due to a distinct color (and shape) differences, by the colorimetric imaging method after ~5 min incubation with bromocresol green. Thus, the agent and approach have the potential for sensitive early cancer diagnosis on cell level and related applications. Compared to our recently reported work on cancer cell screening by using plasmonic glucose nanoprobe^20–22^, this method is much simpler and more effective.

### Single-cell pH accurate detection and profiling

**Figure 3:**
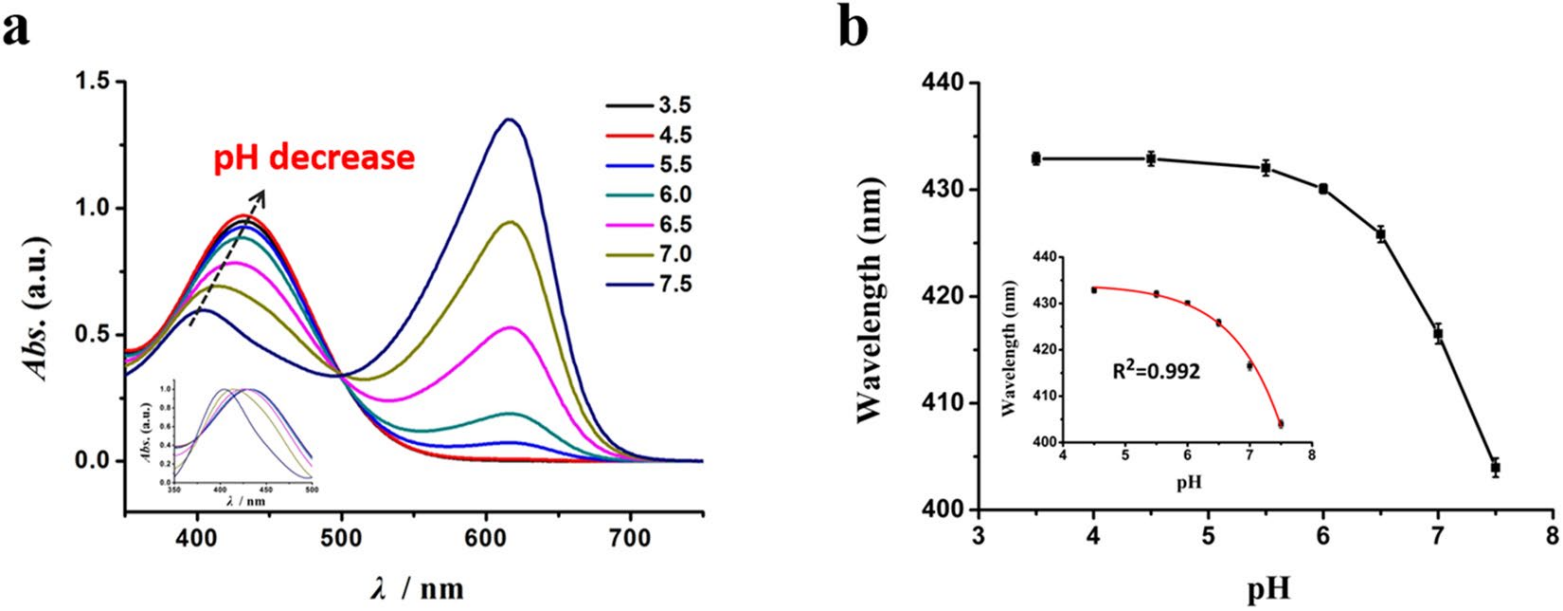
UV-Vis absorption spectra and calibration curve of bromothymol blue/PBS solutions with different pH values. (**a**) Traditional UV-Vis absorption spectra of bromothymol blue/PBS solutions (10 mM) with varied pH values. The inset is the normalized UV-Vis absorption spectra of 350–500 nm for bromothymol blue/ PBS solutions (10 mM) with varied pH values. (**b**) The calibration curve of bromothymol blue solutions in terms of the pH-dependent UV-Vis absorption peak for pH measurements. Inset shows the calibration curve from pH 4.5 to 7.5. The error bars illustrate the relative standard deviation (RSD) over three replicates.

Single-cell pH detection was further achieved by our UV-Vis microspectroscopic method, using bromothymol blue as a pH indicator and collecting the absorption spectra on a single-cell level. First, we studied the absorption spectra of bromothymol blue PBS solutions with varied pH values to obtain the correlation for quantitative pH detection of single-cells. There are two obvious features in the spectra (Fig. 3a). One is the absorption peak centered around ~400–440 nm, which is highly pH-dependent and will turn stronger and red-shift distinctly from 403 nm to 433 nm (and reaches saturation thereafter) as the solution pH value decrease from 7.5 to 4.5. Another feature is that a fixed absorption peak centered at 616 nm appears and the peak intensity decreases gradually and finally disappears when solution pH turns acidic. This peak can be attributed to the occurrence of hydrogen transference (on the phenolic groups) and intramolecular charge transfer (caused by the conjugate effects of the system)^23, 24^, which is complicated in cell systems and susceptible to errors and therefore not applicable for quantitative single-cell pH analysis in this study. Figure 3b shows the plot of the first absorption peak as a function of pH for the bromothymol blue solutions with pH values varied from 3.5 to 7.5, with error bars derived from three replicates. The inset in the Fig. 3b is the fitted nonlinear calibration curve for pH ranges of 4.5–7.5 with R^2^ = 0.992.

**Figure 4:**
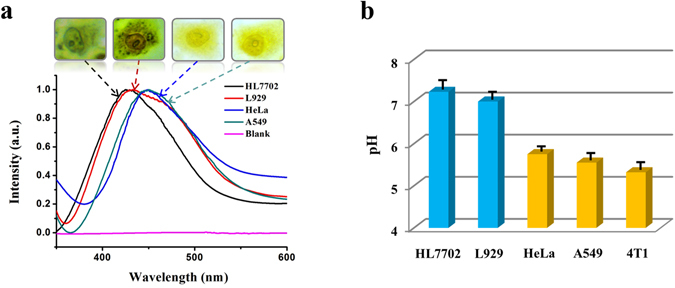
Single-cell pH accurate detection and profiling by the microspectroscopy-based UV-Vis colorimetric method. (**a**) Normalized bright-field microscope-based single-cell absorption spectra and corresponding single-cell microscope images of HL7702, L929, HeLa, and A549 cells recorded by our UV-Vis microspectroscopy, after the treatment with bromothymol blue. (**b**) Histogram of the pH distribution for single HL7702, L929, HeLa, A549, and 4T1 cells, respectively, detected by the method with error bars based on at least twenty single cells for each cell line.

Typically the normalized bright-field optical microscope-based single-cell absorption spectra and corresponding single-cell microscope images of HL7702, L929, HeLa, and A549 cells were recorded by the method (Fig. 4a), using bromothymol blue as a pH indicator and randomly selected bare A549 single cells (indicator-free) as a blank sample. Here, the single-cell absorption spectra were all recorded solely from an intact single cell (acting as a sample container) and used to obtain the averaged pH value of a single whole-cell. The UV-Vis absorption peaks of single HL7702, L929, HeLa, and A549 cells were clearly observed without interference from the intracellular substances of the cell. The peaks were distinguishable with a gradual red-shift, which means that their pH values were decreased gradually according to Fig. 3a. The results were quite reproducible for each cell type, making the method feasible and reliable for accurate single-cell pH detection. Incidentally, we found experimentally that there is always a fixed ~17 nm red-shift deviation of the indicator absorption peak obtained by the microscope-based spectroscopic method (Fig. S5) as compared with that taken by traditional UV-Vis spectrometer, which may stem from the differences in methodology and the surface-confinement effects that may exist in the microscopic method. Therefore, for accurate single-cell pH detection the absorption spectra of the bromothymol blue-dyed single-cells were corrected accordingly. Figure 4b shows the histogram of the pH distribution for single HL7702, L929, HeLa, A549, and 4T1 cells, respectively, detected by the method after the calibration. For each cell type, we detected the absorption spectra of at least twenty randomly selected single cells to ensure reproducibility and reliability of the method. As shown in Fig. 4b, the diversity of cell pH for different cell types was revealed, with the averaged pH values of single HL7702, L929, HeLa, A549, and 4T1 cells in this study measured to be 7.23 ± 0.2, 7.0 ± 0.18, 5.75 ± 0.10, 5.5 ± 0.15, and 5.32 ± 0.13, respectively. The measured average pH value of the intact single HeLa cell is very consistent with the previously reported value (5.7 ± 0.2)^5^.

### Cancer-cell identification from cocultured healthy cells by pH imaging and accurate detection

**Figure 5:**
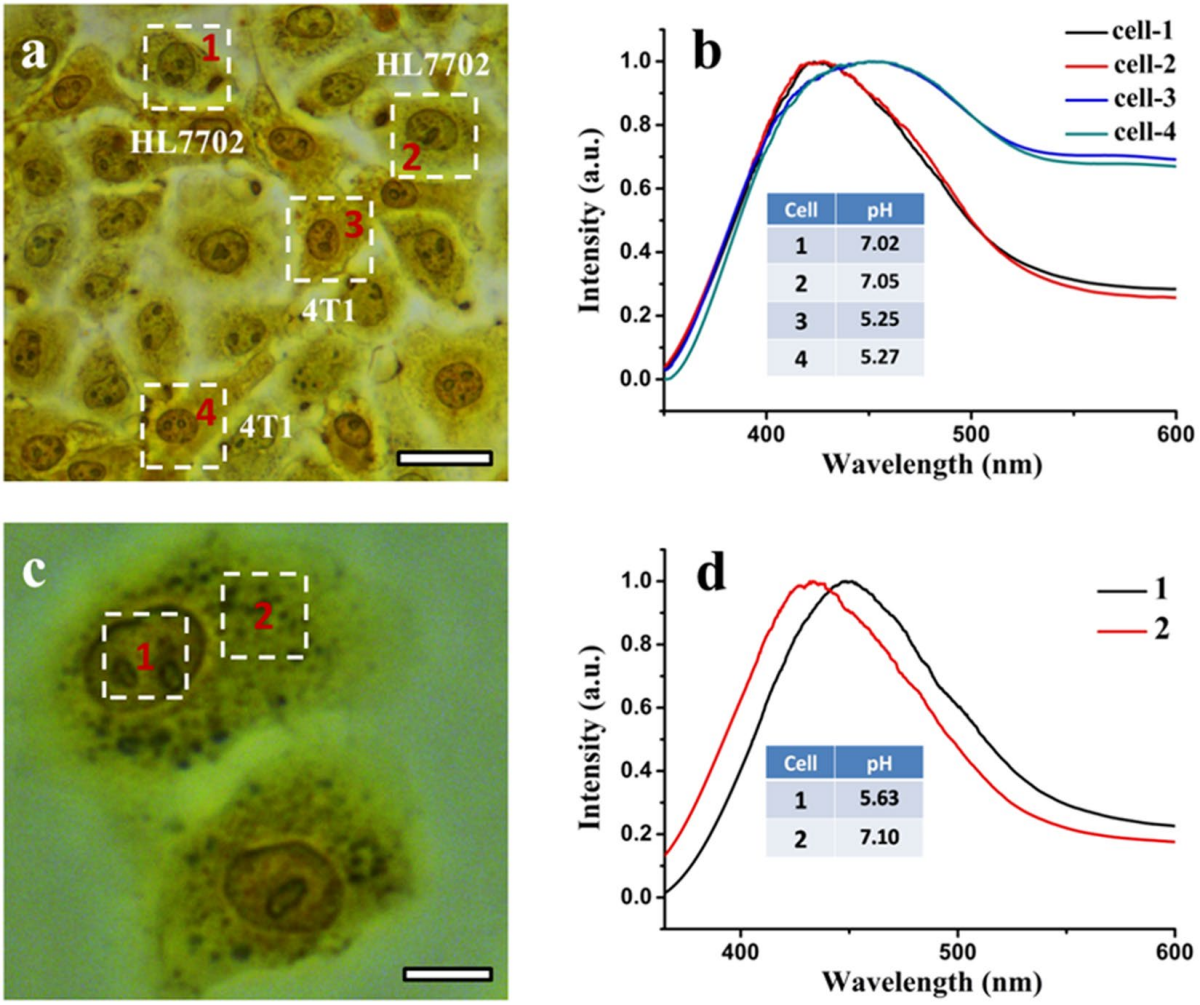
Cancer cell identification from cocultured normal cells by pH imaging and detection, and sub-cellular pH detection. (**a**) A bright-field optical microscope image of the cocultured HL7702 and 4T1 cells treated with bromothymol blue. Four intact single cells (with corresponding spectrum acquisition area marked by a dotted white square) were randomly selected for single-cell pH detection. Scale bar: 20 μm. (**b**) Corresponding normalized bright-field microscope-based single-cell absorption spectra of the four selected cells as denoted in (**a**). (**c**) A bright-field optical microscope image of 1L929 cells treated with bromothymol blue. A single L929 cell marked by two dotted white squares (in different parts of the cell) was selected for sub-cellular pH detection. Scale bar: 10 μm. (**d**) Corresponding normalized sub-cellular absorption spectra of the L929 cell taken from the two different parts of the cell as denoted in (**c**).

We further examined the feasibility of the single-cell pH detection method for potential clinical applications. A coculture of cancerous 4T1 and normal HL7702 cells (ratio of 1:1) was prepared and tested. Figure 5a and 5b display a bright-field optical microscope image and corresponding single-cell UV-Vis absorption spectra, taken from the four randomly selected single-cells as denoted in Fig. 5a, of the cocultured cells treated with bromothymol blue. The absorption spectra taken from cell 1 and cell 2 are almost identical, so is the case for cell 3 and cell 4. The spectra as well as the calculated pH values (as shown in the inset of Fig. 5b) were in good accordance with those taken from individually cultured cell samples (cf. spectra for HL7702 and 4T1 cells in Fig. 4a and corresponding pH distributions in Fig. 4b). Therefore, the cancerous 4T1 cells can be easily identified from the cocultured cells. This means that the developed method can be applied for label-free rapid identification of cancer cells from cocultured normal cells as they have different distinguishable intracellular pH levels.

### Sub-cellular pH detection

Finally, we also examined the possibility of the method for sub-cellular pH detection. In this study, we randomly chose L929 cells as a sample. Figure 5c shows the bright-field microscope image of a bromothymol blue-dyed L929 cell marked by two dotted white squares (in the nucleus and cytoplasm part of the cell, respectively), which was selected for sub-cellular pH detection. Figure 5d shows the corresponding selected-area sub-cellular absorption spectra of the cell. As clearly seen from the figure, the absorption peak of the cell taken mainly from the nucleus has a pronounced red-shift (~16 nm) compared with that taken from the cytoplasm part, from which the pH values of the nucleus and cytoplasm part of the cell were calculated to be 5.63 and 7.10, respectively. This is also a strong indicator that the measured pHs are responding to the intracellular environment rather than extracellular milieu. If the indicators are localized to the cell membrane, the absorption peak of the two parts should be the same. Also, the pH measurements in this study are quite reproducible and the measured pH values varied only between different cell line, which can further exclude the possibility that the pH indicators are being affected more by the extracellular environment than the intracellular environment.

## Discussion

We have successively developed a simple and effective agent (and method) by using bromothymol blue or bromocresol green, which are commonly be used for solution pH measurements, for living-cell imaging as well as single-cell intracellular pH sensing and profiling for the first time, and succeeded in using this method for cancer cell identification by combined use of bright-field microscopy. After incubating the pH indicators with living cells for a certain period of time, the outlines, even the nucleus and cytoplasm of the cells can be easily distinguished, it means that the pH indicator can be used as a new and effective cell-staining agent for living cell imaging. For the experiments, the newly-prepared bromothymol blue or bromocresol green was immediately incubated with the cells after removed the culture medium and rinsed three times with 20% ethanol aqueous solution. From Fig. 2a, we can visually identify cancer cells from health normal cells in a facile and fast manner. The living-cell imaging and pH-profiling experiments are quite reproducible. We repeated each experience for more than 3 times all had similar results. More importantly, as shown in Fig. 2c, tumor cells can be easily visually identified from co-cultured normal health cells (which simulates a clinical cell sample), making the pH indicators and microscopy-based approach very promising for potential clinical use. Besides, as can be seen from Figs 3 and 4, the pH values of different kind of cells can be detected by combined use of pH indicators and the bright-field microscope-based single-cell UV-Vis microspectroscopy. Meanwhile, this method can be applied for the rapid identification of cancer cells from cocultured normal cells due to their different distinguishable intracellular pH levels (Fig. 5a and b). Also, it can be used in the selected-area sub-cellular pH detection, because different parts of a single cell have different pH levels and represented different microscope-based absorption spectra (Fig. 5c and d).

It is noted that although acid-extrusion by active transport is important in metabolically active cancer cells, where it removes excess intracellular acid and sets the intracellular resting pH^7^, the exact intracellular pH levels (acidic or basic than normal cells) of cancer cells are still debated due to the lack of a direct and reliable method to detect the pHs of single cells, especially for the pH of the nucleus^13^. Most researchers assume that the nuclear pH equals that of the cytosol, which is based on an assumed mechanism that requires experimental validation^13^. The direct, reliable (sub)cellular pH detection method we developed herein may provide a strong single-cell evidence to help solving such controversy, and will extend the method for exploring interesting and important sub-cellular pH-related studies.

Up to now, cancer has become one of the most serious global health threats, and the morbidity is rising. More and more methods have been used as powerful tools for clinical detection of cancer, including magnetic resonance imaging (MRI), currently computed tomography (CT), immunofluorescence assay and pathological immunohistochemistry (IHC), protein chip technology and so on. However, these methods always suffer from some disadvantages, such as too expensive to perform them on all suspect patients, low positive predictive value, high individual variation and time-consuming procedures. In addition, they are still not sensitive enough for the early diagnosis of cancer, especially on single cell level. Due to simplicity, rapidity, effectivity and availability, our pH indicators and the bright-field microscopy imaging technique would be promising for early detection of cancers, especially for high-throughput *in-vitro* cancer preliminary visual screening of cell samples for suspect patients.

In conclusion, we report a simple and reliable colorimetric method for single-cell pH imaging and sensing by combined use of UV-Vis microspectroscopy and two common pH indicators. Accurate and sensitive intracellular pH profiling and detection on single-cell or sub-cell level with good reproducibility has been achieved by the method, which enables facile pH discrimination and label-free rapid identification of cancer-cells from normal cells (due to distinguishable intracellular pH levels). The practicality of the proposed approach was further simply illustrated by its successful application to discriminate cancer cells from cocultured normal cells as they have distinct different intracellular pH levels. The proposed small molecule cell pH-sensing probe and approach, which based on the use of noncytotoxic common agents and bright-field microscope and possessing only a very simple one-step process, are much simpler and effective than the previously reported methods that usually need complicated nanoprobe preparations and lack reproducibility. This work provides a new and reliable method for accurate intracellular pH-sensing and –profiling, and may open a new avenue for pH-related single-cell studies, which are important and will promote the understanding of the physicochemical properties of cells, especially cancer cells. We also envision that the combined use of the microspectroscopy-based single-cell research platform and smart/multifunctional plasmonic nanoprobes^21, 22, 25, 26^ will benefit and enrich single-cell studies. The work also inspires the development of new agents and effective tools for early cancer diagnosis on single-cell level.

## Materials and Methods

### Materials

Sodium chloride (NaCl), potassium chloride (KCl), disodium hydrogen phosphate (Na_2_HPO_4_), potassium phosphate monobasic (KH_2_PO_4_) and hydrochloric acid (HCl) were purchased from Beijing Chemical Corp. Bromothymol blue, bromocresol green, methyl red and thymol blue were obtained from Aladin Ltd. (Shanghai, China). 3-(4,5-Dimethylthiazol-2-yl)-2,5-diphenyltetrazolium bromide (MTT) was purchased from Boster Biological Technology Co, Ltd. Dimethyl sulfoxide (DMSO) was obtained from Xilong Chemical Industry Incorporated Co, Ltd. Eagle’s medium and Fetal bovine serum were purchased from Gibco. Penicillin and streptomycin were obtained from Invitrogen. All of the chemicals were used as received and without any further purification. All glassware and stirrer bars were cleaned extremely carefully in aqua regia (3:1 v/v HCl (37%): HNO_3_ (65%)) solution (Caution: aqua regia solutions are extremely corrosive and should be handled with extreme care, gloves and eye protections are required for handling. Never store these solutions in closed containers) and then rinsed thoroughly with H_2_O before use. All the aqueous solutions were prepared using deionized (DI) water. The water was prepared in a three-stage Millipore Milli-Q purification system and had a resistivity higher than 18.2 MΩ cm.

### Cell Culture

HeLa, HepG2, A549, HL7702, L929, and 4T1 cells were obtained from the American Type Culture Collection (ATCC, USA). The cells were maintained in Dulbecco’s modified Eagle’s medium supplemented with 10% fetal bovine serum which contains 200 units mL^−1^ streptomycin and 200 units mL^−1^ penicillin. All of the cells were cultured in a plastic cell culture dish maintained in culture flasks at 37 °C in a humidified atmosphere containing 5% CO_2_. The number of cells was counted to be ca. 5 × 10^6^ cells mL^−1^.

### Cell Staining and pH-Profiling with pH Indicators

For the cell staining and pH-profiling, the cell culture medium was first removed and then washed the cells with 20% ethanol aqueous solution for three times. Next, pH indicator solution (1 mg mL^−1^) was added to the plate and removed after interacting with the cells for ~5 min. Finally, the cells were washed with 20% ethanol aqueous solution again for three times to remove the redundant pH indicator.

### Cell Viability study

In order to assess the cytotoxicity of the bromothymol blue and bromocresol green, the standard methyl thiazolyltetrazolium (MTT) assay was carried out to determine relative cell viabilities after 24 h treatments with bromothymol blue or bromocresol green. The absorbance of formazan (produced by the cleavage of MTT by dehydrogenases in living cells) is directly proportional to the number of live cells^27^. For each MTT assay, 96 culture wells of A549, HeLa, HL7702, and L929 cells were prepared (~5000 cells/well). After the cells attached to the plate, the cell culture medium was sucked out, and then 0.1 ml of bromothymol blue (1 mg mL^−1^) or bromocresol green (1 mg mL^−1^) were added to each kind of cells and incubated for ~10 min, respectively. Next, the pH indicators were sucked out and the culture medium (0.1 mL) was re-added to the 96-well assay plates. After 24 h of incubation, 10 μL of the MTT solution was added to each well of the 96-well assay plates and thoroughly mixed. Four hours later, 100 μL of DMSO was added to each well to lyse the cells. The complete assay was performed at least three times, and the results were averaged. The cell viability was calculated as the ratio of the absorbance of the sample well to that of the control well and expressed as percent viability, assigning the viability of nontreated cells as 100%.

### Microscope-Based Colorimetric Cell Imaging and Single-Cell pH Detection

Microscopic and bright-field images were obtained with a DMI6000 B inverted microscope (Leica) and a DFC450 digital color camera (Leica). Prior to imaging, the cell culture medium was removed and then the cells were washed with 20% ethanol aqueous solution for three times. Next, the pH indicator solution was added to the plate and removed after interacting with the cells for ~5 min. Finally, the cells were washed with 20% ethanol aqueous solution again for three times to remove the redundant pH indicator. For the MTT assay, absorbance values of the formazan were detected by Bio-Rad model-680 microplate reader at 570 nm. To collect the absorption spectra of a single cell, a Spectrasuite spectrometer with a small collecting spot (300 × 300 μm) was used. The reference spectrum was collected from a clean area where no cells or contaminants were presented, and then was subtracted and used to normalize the sample absorption spectra.

## Electronic supplementary material

supporting information for publication
