# Supplementary material for: Single-cell pH imaging and detection for pH profiling and label-free rapid identification of cancer-cells
Source: Sci Rep. 2017 May 11;7:1759. doi: 10.1038/s41598-017-01956-1 (PMC5431805; doi:10.1038/s41598-017-01956-1)
Supplement: Supplementary file 1 — supporting information for publication [file 41598_2017_1956_MOESM1_ESM.pdf]

Supporting information

Single-cell pH imaging and detection for pH profiling and label-free rapid identification of cancer-cells

Hui Hou[1, 2], Yangyang Zhao[1], Chuanping Li[1, 2], Minmin Wang[1, 2], Xiaolong Xu[1] and Yongdong Jin*[,1]

[1]State Key Laboratory of Electroanalytical Chemistry, Changchun Institute of Applied Chemistry, Chinese Academy of Sciences, Changchun 130022, Jilin, China.

[2]University of Chinese Academy of Sciences, Beijing100049, China. Correspondence and requests for materials should be addressed to Yongdong Jin (email: ydjin@ciac.ac.cn)

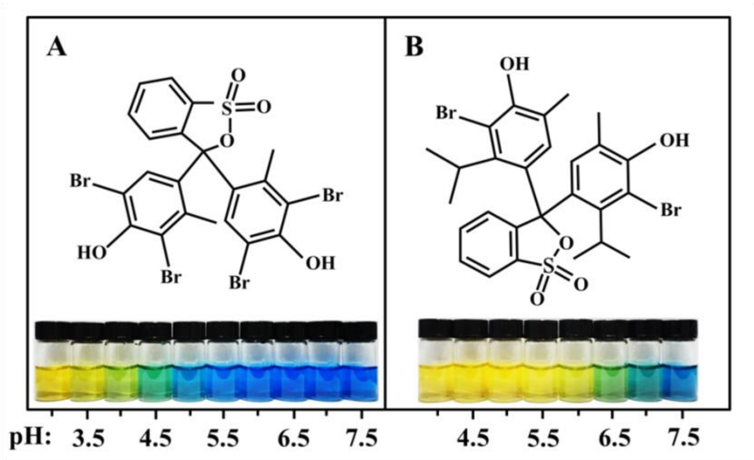

Fig. S1. The chemical structures of (A) bromocresol green and (B) bromothymol blue. The insets at the bottom show the digital photographs of the corresponding bromocresol green/ and bromothymol blue/ PBS solutions for different pH values.

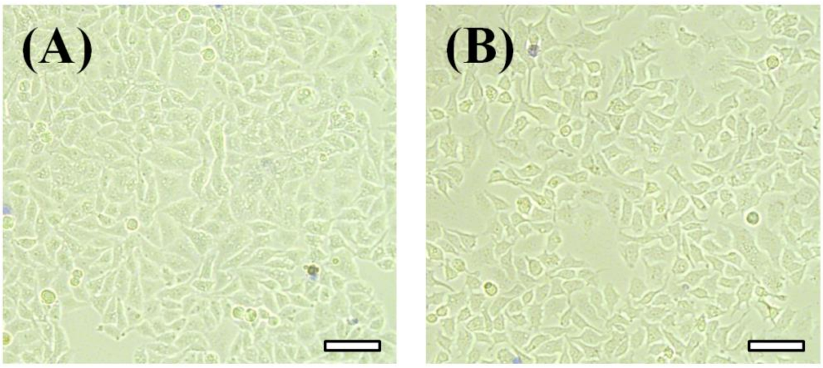

Fig. S2. Bright field optical microscope images of (A) HeLa and (B) L929 cells after the incubation with 20％ ethanol solution for 10 min, all the cells were stained with trypan blue dye to examine the level of cell damage. Scale bar: 50μm.

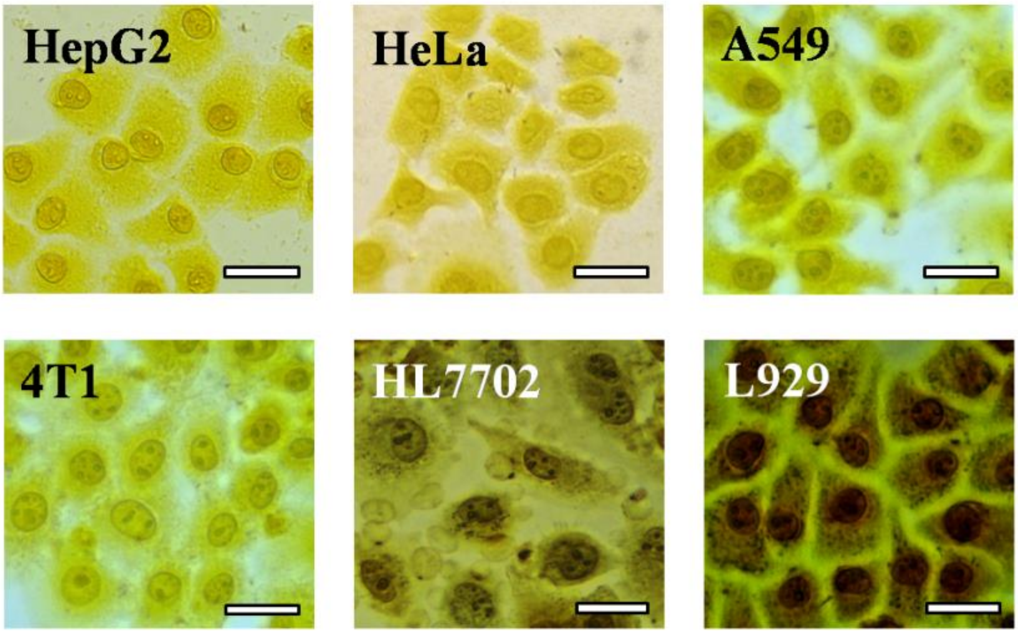

Fig. S3. Bright field optical microscope images of cancerous HepG2, HeLa, A549, 4T1 cells, and normal HL7702 and L929 cells after the incubation with bromocresol green, respectively, for 5 min. All scale bars: 20 μm. The cancerous acidic HepG2, HeLa, A549 and 4T1 cells were bright yellow colored after the treatment with bromocresol green, whereas the normal HL7702 and L929 cells (with neutral intracellular environment) were stained with blue. The tested cancer cells can also be easily discriminated from normal healthy cells by using bromocresol green as a cell staining agent.

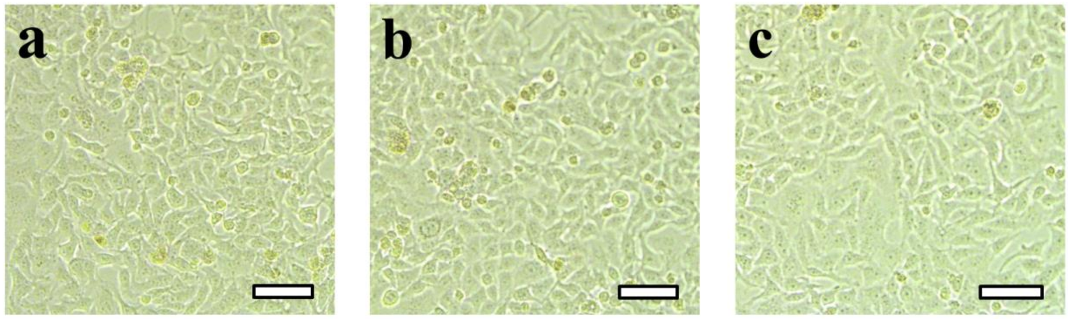

Fig. S4. Bright-field microscope images of HeLa cells before (a) and after treatment with methyl red (b) and thymol blue (c). All scale bars: 50 μm.

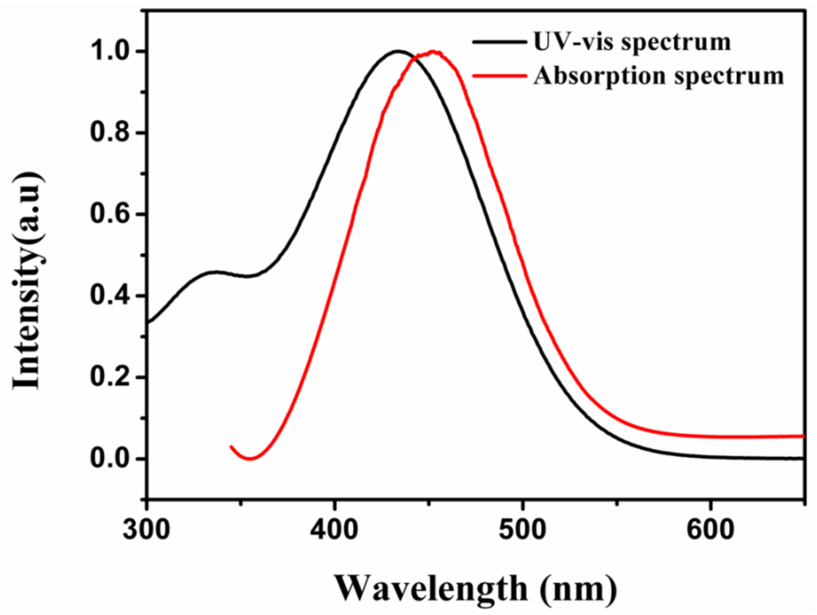

Fig. S5. Typical UV–Vis absorption spectra of the same bromothymol blue solution sample with pH value of 3.5, obtained by traditional UV-Vis spectrometer (black) and the microscope-based spectroscopic method (red), respectively.
